# Consistent responses to moisture stress despite diverse growth forms within mountain fynbos communities

**DOI:** 10.1007/s00442-023-05326-9

**Published:** 2023-01-24

**Authors:** Robert P. Skelton, Adam G. West, Daniel Buttner, Todd E. Dawson

**Affiliations:** 1 SAEON Fynbos Node, Cape Town, South Africa; 2 Department of Biological Sciences, University of Cape Town, Cape Town, South Africa; 3 Department of Botany, Nelson Mandela University, Gqeberha, South Africa; 4 Department of Integrative Biology, University of California, Berkeley, Berkeley, USA

**Keywords:** Drought, Ecophysiology, Plant water use, Sap flow, Mountain fynbos

## Abstract

Understanding climate change impacts on the Cape Floristic Region requires improved knowledge of plant physiological responses to the environment. Studies examining physiological responses of mountain fynbos have consisted of campaign-based measurements, capturing snapshots of plant water relations and photosynthesis. We examine conclusions drawn from prior studies by tracking in situ physiological responses of three species, representing dominant growth forms (proteoid, ericoid, restioid), over 2 years using miniature continuous sap flow technology, long-term observations of leaf/culm water potential and gas exchange, and xylem vulnerability to embolism. We observed considerable inter-specific variation in the timing and extent of seasonal declines in productivity. Shallow-rooted *Erica monsoniana* exhibited steep within-season declines in sap flow and water potentials, and pronounced inter-annual variability in total daily sap flux (*J*_s_). *Protea repens* showed steady reductions in *J*_s_ across both years, despite deeper roots and less negative water potentials. *Cannomois congesta*—a shallow-rooted restioid—was least negatively impacted. Following rehydrating rain at the end of summer, gas exchange recovery was lower in the drier year compared with the normal year, but did not differ between species. Loss of function in the drier year was partially accounted for by loss of xylem transport capacity in *Erica* and *Cannomois*, but not *Protea.* Hitherto unseen water use patterns, including inter-annual variability of gas exchange associated with contrasting water uptake properties, reveal that species use different mechanisms to cope with summer dry periods. Revealing physiological responses of key growth forms enhances predictions of plant function within mountain fynbos under future conditions.

**Supplementary Information:**

The online version contains supplementary material available at 10.1007/s00442-023-05326-9.

## Introduction

Drought events in natural plant communities have the potential to alter plant community composition and function by differentially impacting species mortality (Anderegg et al. 2015; Choat et al. 2018; McDowell et al. 2020; Hammond et al. 2022). However, in addition to extreme droughts that cause extensive plant die-off, there are many droughts that do not cause mortality, but instead cause prolonged reductions in plant transpiration and primary production, both during the dry period and after rainfall returns (West et al. 2008; Anderegg et al. 2015; Garcia-Forner et al. 2016; Skelton et al. 2017; McDowell et al. 2020). While non-lethal droughts may not result in visible plant mortality, they may still cause considerable loss of function in plants, which may influence resilience to additional stressors and alter community dynamics if species are differentially affected.

Estimating the extent of the loss of function caused by droughts in natural plant communities requires robust mechanistic frameworks of plant response to the environment that are based on quantitative physiology (Oren et al. 1999; Jackson et al. 2000; Sperry 2000; Plaut et al. 2012; Martínez-Vilalta et al. 2014; Skelton et al. 2015). South Africa’s Cape Floristic Region (CFR) is one of the smallest global biodiversity hotspots, containing over 8500 species in an area of approximately 90,000 km^2^ (Allsopp et al. 2014). The combination of high species richness, high environmental heterogeneity, and strong interactions between climate and plant diversity (Cowling et al. 2005; Procheş et al. 2005) makes the CFR an ideal location to examine plant functional responses (West et al. 2012; Allsopp et al. 2014; Altwegg et al. 2014).

Previous work in mountain fynbos—a dominant vegetation type in the CFR—has revealed the key role of growth form, rooting depth, and stomatal dynamics in determining plant exposure to drought with implications for productivity. For example, deep-rooted proteoids—the dominant overstorey shrubs—have been found to exhibit little or no moisture stress throughout the year (Moll and Sommerville 1985; van der Heyden and Lewis 1989; West et al. 2012) and have shown no photosynthetic responses to irrigation (e.g. van der Heyden and Lewis 1990; Herppich, et al. 1994). Small- to medium-sized shallow-rooted shrubs (ericoids) tend to display low seasonal leaf water potentials (Miller et al. 1983, 1984; Higgins et al. 1987; van der Heyden and Lewis 1989; West et al. 2012), although they too have shown no photosynthetic response to irrigation (van der Heyden and Lewis 1990). Water potentials in restioids—reed-like monocotyledons—tend to remain relatively high (Miller et al. 1984; West et al. 2012), although seasonal declines have been observed in some species (e.g. Moll and Sommerville 1985; van der Heyden and Lewis 1989) and irrigation during summer has resulted in a 20–40% increase in net photosynthetic rate in one study species (van der Heyden and Lewis 1990). Furthermore, recent work has shown limited plasticity in response to experimental changes in seasonal moisture availability in fynbos, particularly in established shrubs (van Blerk et al 2021a, b).

However, most prior studies examining physiological responses of mountain fynbos species have consisted of campaign-based measurements capturing snapshots in time of plant water relations and photosynthesis (Miller et al. 1983, 1984; Moll and Sommerville 1985; von Willert et al. 1989; van der Heyden and Lewis 1989, 1990; Herppich, et al. 1994; West et al. 2012). While highly informative, snapshots may be influenced by preceding conditions that may obscure true patterns and drivers of plant function. Although the south-western corner of Southern Africa is traditionally thought of as a winter-rainfall system, rain-bearing frontal systems occur throughout the year. Thus, unlike other global regions with Mediterranean-type climates (e.g. California or the Mediterranean Basin), it is rare to go longer than 2 weeks without some measurable precipitation (Richardson and Kruger 1990; Cowling et al. 2005; Agenbag et al. 2008; Arnolds et al. 2015).

Additionally, clouds provide considerable moisture inputs during summer that could lessen plant moisture stress between rainfall events (Marloth 1905; Nagel 1956). During the summer months, rainfall events are often preceded by hot, dry, and windy conditions resulting from northerly airflow from the interior of Southern Africa. These opposing systems result in strongly pulsed atmospheric and soil moisture conditions that may vary over a matter of days, and mean that snapshot campaign measurements may miss the full picture of dynamic plant physiology in this region. To fully understand plant water use and foliar gas exchange in such a highly pulsed environment and to decouple the effects of short-term inputs versus longer-term trends, it is necessary to monitor these processes over multiple years.

The objective of this study was to quantify in situ physiological responses within mountain fynbos of the CFR to identify patterns of plant response to dry conditions and enhance our understanding of the potential impacts of drought on this system. Despite the known generalities of plant response to drought found within these systems, several questions remain unresolved. For instance, the precise timing of physiological responses within a drought period (e.g. stomatal closure) may vary between species, with unclear implications for productivity and coexistence (West et al. 2012; Skelton et al. 2015). Further, physiological tolerance limits, such as xylem vulnerability to embolism, may also vary between species, ensuring that dry periods can have varying impacts on species (Jacobsen et al. 2009; Paddock et al. 2013).

In this study, we tracked in situ physiological changes in three sample species, representing the three dominant growth forms in the fynbos, over 2 years. To do so, we used miniature external (i.e. non-invasive) sap flow technology (Skelton et al. 2013) in combination with long-term observations of leaf or culm water potential and gas exchange. In addition, we quantified xylem vulnerability to embolism of the three species to evaluate hydraulic safety margins. Our continuous measurements allowed us to examine, in unprecedented detail, the diurnal and seasonal responses of each species, enabling us to achieve a major aim of assessing the accuracy and generality of the snapshot campaign measurements previously captured in the region. Our results reveal that inter-specific, within-season differences in xylem water relations drive variation in the timing and extent of dehydration-induced declines in productivity. However, inter-specific variation in xylem vulnerability to embolism ensures convergence in long-term water transport function under moderately dry conditions.

## Materials and methods

### Study site and species

The field site was situated at Jonaskop in the Riviersonderend Mountains (33° 56ʹ 30.45ʺ S and 19° 31ʹ 34.18ʺ E, 980 m elevation above sea level), located in the south-west corner of Southern Africa’s CFR. The geological substratum is composed mostly of nutrient poor quarzitic sandstone of the Table Mountain Group, which produces shallow, rocky, highly leached sandy soils with low water retention capacity. The site receives 411 mm of rainfall on average annually, approximately 66% of which falls in winter (April–Sept) (Agenbag et al. 2008) (Table S1). Mean annual temperature is 13.6 °C, mean minimum temperature of the coldest month is 3.6 °C and the mean maximum temperature of the warmest month is 27.4 °C (Agenbag et al. 2008). Vegetation at the site is mountain fynbos composed of a 2–3 m tall open canopy dominated by *Protea repens* (L.) L. (Proteaceae) and an understorey of ericoid shrubs and reed-like graminoids (Agenbag et al. 2008) (Fig. S1). Three species were selected for detailed physiological monitoring: *Erica monsoniana* L.f. (Ericaceae) is a small- to medium-sized, small-leaved woody ericoid shrub; *Protea repens* is a broad-leaved, woody proteoid shrub; and *Cannomois congesta* Mast. (Restionaceae) is a reed-like rhizomatous perennial with erect, lignified culms typical of the restioid growth form (Fig. S1). The three morphologically dissimilar species are common in the community and display growth forms that are typical of the three most dominant functional types in mountain fynbos. In addition, the species are either widespread across the CFR (*Protea repens*) or are a representative species from a widespread and abundant genus (e.g. *Erica* and *Cannomois*).

### Micrometeorological monitoring station

A micrometeorological station erected at the study site in 2011 (described previously in Skelton et al. 2013) monitored environmental variables continuously throughout the study period. The micrometeorological station was located on a level, slightly north-facing plateau and all measured plant individuals were located within a zone of approximately 100 m radius from the station. Briefly, the micrometeorological station consisted of a 3 m tall tripod (CM106, Campbell Scientific, Logan, Utah, USA) and sensors, including a temperature and relative humidity probe (HMP45C, Campbell Scientific), a leaf-wetness sensor (237, Campbell Scientific), a tipping-bucket rain gauge, and a soil water content profile probe (EnviroSMART, Campbell Scientific). Meteorological variables were logged at half-hour intervals (CR1000, Campbell Scientific). Dew events were defined as periods without rainfall when the resistance of the leaf-wetness sensor fell below 400 kOhms and the air temperature (*T*_air_, ℃) was within 0.5 ℃ of the dew point temperature (*T*_dew_point_, ℃), calculated as follows:

1 $$ T_{{\text{dew-point}}} = B \left[ {\ln \left( {{\text{RH}}/100} \right) + \, AT_{{{\text{air}}}} / \, \left( {B \, + \, T_{{{\text{air}}}} } \right)} \right]/\left( {A - \ln \left( {{\text{RH}}/100} \right) - \;(A T_{{{\text{air}}}} )/\left( {B + T_{{{\text{air}}}} } \right)} \right), $$

where RH is the relative humidity (%), *A* = 17.625 and *B* = 243.04 ℃ (Lawrence 2005).

To calibrate the soil moisture probe we compared the output to soil water content assessed gravimetrically. Soil samples were collected periodically throughout the study period using an auger at sites located approximately 1 m from the soil moisture probe and at a depth of 50 cm. Soil samples were placed in vials and then sealed before being weighed, placed in a drying oven at 70 °C for 48 h, and then weighed again. The relationship between gravimetric soil moisture content (in grams of H_2_O per gram of dry soil) and probe output was strong, linear, and highly significant (slope = 1.53, intercept =  − 1.54, *R*^2^ = 0.94, *p* < 0.00002).

### Leaf gas exchange and water potentials

Predawn and midday leaf or culm water potentials (MPa) were measured on shoots or culms of at least five individuals of each species at least once every month, from February 2012 until May 2014, using a Scholander pressure chamber (PMS Instruments, Corvallis, OR, USA). Measurements were made more frequently during periods of water stress, or when plants exhibited rapid changes in leaf water potential (e.g. following rainfall). Predawn measurements were made approximately 1 h before sunrise, and midday measurements were made between 12:00 and 14:00 on the same five individuals of each species. Measurements were made on different individuals within the community at each sampling date due to the destructive nature of the sampling.

Leaf or culm carbon assimilation (*A*, µmol m^−2^ s^−1^) and stomatal conductance (*g*_s_, mol m^−2^ s^−1^) were measured using an infrared gas analyser (Li-Cor 6400; Li-Cor BioSciences, Lincoln, NE, USA). Measurements were taken concurrently with the midday water potentials and on leaves or culms from the same individuals. Light intensity in the cuvette was set at 1500 μmol m^−2^ s^−1^, humidity was maintained slightly below (< 0.2 kPa) ambient, and reference CO_2_ concentration was held at 400 mmol m^−2^ s^−1^. Leaf or culm temperature was maintained between 25 and 33 °C as calculated using energy balance equations to track ambient conditions.

### Sap flow

Sap flow was monitored for two summers (Oct–Mar) in at least five individuals of each species. Sap flow was measured using miniature external (i.e. non-invasive) heat ratio method (HRM) gauges described in Clearwater et al. (2009) and Skelton et al. (2013). Sap flow gauges were re-installed on each individual at the start of each new monitoring season (in spring) to avoid wounding or growth-mediated effects on the sap flow signal. Sap flow gauges were installed on different individuals to those that were sampled periodically for leaf or culm measurements to minimise any negative impact of repeat destructive sampling. However, age or size-related differences of reseeder species are minimal within fire-prone mountain fynbos communities due to post-fire germination resulting in uniform age cohorts. Heat ratio values were quantified every half-hour for the duration of the season and converted to heat pulse velocity (*v*_h_, cm s^−1^), sap velocity (*v*_s_, cm s^−1^) and sap flux density (*J*_s_, g cm^−2^ s^−1^) using equations and parameters from Skelton et al. (2013). 

### Sap flow-derived stomatal conductance

Sap velocity was converted to sap flow-derived transpiration rate (*E*_sf_, mmol m^−2^ s^−1^) using the relationship between sap velocity and transpiration (*E*, mmol m^−2^ s^−1^) measured on leaves or culms of adjacent individuals (see “Leaf gas exchange and water potential” above). The relationships between midday v_h_ and midday E measured on leaves or culms were both strong and highly significant in all individuals (Table S2). Sap flow-derived stomatal conductance of sunlit leaves or culms, *G*_sf_ (mmol m^−2^ s^−1^), used as a proxy for stomatal conductance of sunlit leaves or culms, was calculated using the relationship between sap flow-derived *E*_sf_ and vapour pressure deficit (VPD, kPa). G_sf_ can be determined from *E*_sf_ and VPD in vegetation types with high aerodynamic conductance and low decoupling coefficient (for a full discussion see Hogg & Hurdle 1997), which applies to the open vegetation in this study:

2 $$ G_{{{\text{sf}}}} = \left( \alpha \right)E_{{{\text{sf}}}} /{\text{VPD,}} $$

where α is atmospheric pressure of water vapour equal to *ρ*_*w*_ *G*_*v*_ *T*; *ρ*_*w*_ is the density of water (*c*. 1000 kg m^−3^); *G*_*v*_ is the universal gas constant for water vapour (= 0.462 m^3^ kPa kg^−1^ K^−1^) and *T* is air temperature (Kelvin).

### Seasonal decline and recovery of plant function

Mesic periods in October, prior to the onset of summer moisture limitation, provided a maximum gas exchange reference period against which to quantitatively evaluate seasonal water use. Leaf or culm stomatal conductance, assimilation rate and predawn water potential of all individuals confirmed that plant functionality during this early season window was at, or close to, a non-stressed maximum of each growing season for all species. Within-season recovery was quantified as the maximum % recovery of *J*_s_ or *G*_sf_ (relative to the October reference period) following a post-summer return to pre-stressed predawn water potentials.

### Xylem vulnerability to embolism

We collected large branches from individuals of *Protea* and *Erica* and entire rhizomes with > 20 culms from individuals of *Cannomois* in December 2020. Samples were collected from 3 + healthy-looking individuals of each species. To avoid a potential artefact associated with cut, open vessels in the woody species we determined vessel length using the airflow method (Ewers and Fisher 1989) and ensured that the cut material used for the construction of the xylem vulnerability curves was longer than the species’ maximum recorded vessel length. Vessels were short in *Protea* (mean maximum vessel length = 21.1 ± 2.1 cm, *n* = 7) and *Cannomois* (15 ± 0.0 cm, *n* = 3), but spanned the length of *Erica* stems (> 50 cm, *n* = 3). To overcome this limitation, we cut each *Erica* stem below the root collar (maximum branch length ~ 2.5 m). Upon excision, branches and rhizomes were placed in large plastic bags with damp paper towels, which were then sealed to prevent further water loss while being transported to the laboratory for processing.

We used the optical method described in Brodribb et al. (2016a, b), Brodribb et al. (2017), and Skelton et al. (2018) to generate xylem vulnerability curves for each species. Full details of the method, including an overview of the technique, image processing, as well as scripts to guide image capture and analysis, are also available at http://www.opensourceov.org. Briefly, as each individual desiccated on a laboratory bench, we used flatbed scanners (Epson perfection V800 or V850 Scanner, Epson America) to generate a time series of images of an exposed section of the xylem within small branches (diameter < 0.5 cm and always current year growth) or culms. Stems and culms were scanned in reflective mode at 4800 dpi every 5 min over a period of a few days, allowing us to detect embolism within the outer few layers of xylem in each sample. The small sizes of the branches reduced the possibility that our method might have missed significant radial variation in embolism within branches, although this possibility cannot be entirely excluded.

As branches or rhizomes dehydrated, we also quantified stem or culm xylem water potential. For the two woody species, we attached a stem psychrometer (ICT International, Armidale, Australia) to each branch at more than 60 cm from the cut end of the main branch. Stem psychrometers were sealed with high vacuum grease (Dow Corning Corp, Midland, MI, USA) to prevent moisture loss and secured in place with Parafilm (Bemis NA, Neenah, WI, USA). Stem xylem water potential was recorded every 20 min for the duration of the scanning process. We verified the accuracy of the stem psychrometer readings for a subset of individuals by periodically measuring leaf xylem water potential using a Scholander-type pressure chamber (PMS Instruments, Corvallis, Oregon, USA). For *Cannomois* we quantified xylem water potential of culms attached to the same rhizome as the scanned culms using a Scholander-type pressure chamber. While each excised culm was being measured, it was wrapped in moist paper towel and placed in a plastic bag to prevent further water loss. Variation among neighbouring culms was slight (always < 0.1 MPa) indicating that individuals were equilibrated.

Upon completion, image sequences were analysed to identify embolism events, seen as changes in the reflection of the stem or culm xylem. Image subtraction of subsequent images conducted in ImageJ (National Institutes of Health, Bethesda, MD, USA) was used to reveal rapid changes in light transmission or contrast produced by each embolism event. Embolism events were thresholded, allowing automated counting of each event using the analyse-stack function in ImageJ. From the thresholded stack of embolism events we could extract a time-resolved count of embolism events (using the timestamp of each image). We then converted the raw embolism counts to a percentage of total pixels embolized, producing a dataset of time-resolved percent embolism. The time-resolved percent embolism data were combined with the xylem water potential timeline to estimate the culm or stem xylem water potential associated with each embolism event. Xylem vulnerability to embolism was recorded as the relationship between percent embolism and xylem water potential (Ψ), and modelled using a sigmoid function:

3 $$ {\text{Percent embolism}} = 100{-}100/\left( {1 + e^{{a(\Psi {-}b)}} } \right), $$

where *a* corresponds to the sensitivity to decreasing water potential (proportional to the slope of the equation) and *b* is the water potential associated with 50% embolism (P_50_, MPa).

### Seasonal loss of function in stems or culms

We predicted the amount of in situ embolism experienced by each species by combining stem or culm vulnerability curve data with the in situ minimum leaf or culm water potentials following similar methods to Skelton et al. (2017). Our aim was to evaluate whether the plants growing at the study site were likely to have surpassed thresholds associated with embolism formation or not throughout the study period. We used minimum predawn water potential values from the field to reduce differences between leaf or culm water potential and tension in the xylem caused by transpiration.

### Statistical analyses

Relationships between total daily *J*_s_ and soil moisture or mean midday VPD were assessed using mixed linear models using the “lmer” function in the “lme4” package in R v.3.0.2 (R Development Core Team, 2014). The full model treated total daily *J*_s_ as a function of three fixed factors (soil moisture or VPD, species and season) and a random factor (species/season/individual). Post hoc analyses were conducted by comparing the full model to alternative models consisting of fewer fixed factors and/or without interactions. This was achieved using ANOVA and Akaike’s information criterion (AIC) in R. In each case, we selected the best fit model as the one with the lowest AIC score that also differed statistically (*p* < 0.05) from the other models. Given the temporal nature of the sap flow data, there might be some bias in the estimates from the linear mixed effect models: Although the estimates for the coefficients should be robust, the linear mixed effects models might underestimate uncertainty in the main effects. Trait–environment associations compared across species and seasons should be more robust than estimates of associations within a species and season.

We assessed inter-specific and seasonal (i.e. inter-annual) differences in plant physiological variables (e.g. recovery of total daily *J*_s_ and *G*_sf_, predawn and midday leaf or culm water potential) and traits (e.g. P_50_) using ANOVA. This was done using the “aov” function in the “lme4” package in R v.3.0.2. Minimum predawn and midday leaf or culm water potential were expressed as functions of species and season including a species × season interaction term. Recovery of total daily *J*_s_ or of *G*_sf_ was also initially expressed as a function of species and season including a species × season interaction term, with individuals treated as a random effect (intercept only). Post hoc analyses were conducted by comparing the full model to alternative models consisting of fewer fixed factors and/or without interactions and the best fit model was selected as the one with the lowest AIC score that also differed statistically (*p* < 0.05) from the other models. P_50_ was expressed as a function of species only. For the ANOVA tests, when significant main effects were established (*p* < 0.05), post hoc pairwise comparisons were made using Tukey’s multiple comparisons of means tests.

## Results

### Micrometeorological conditions

**Fig. 1:**
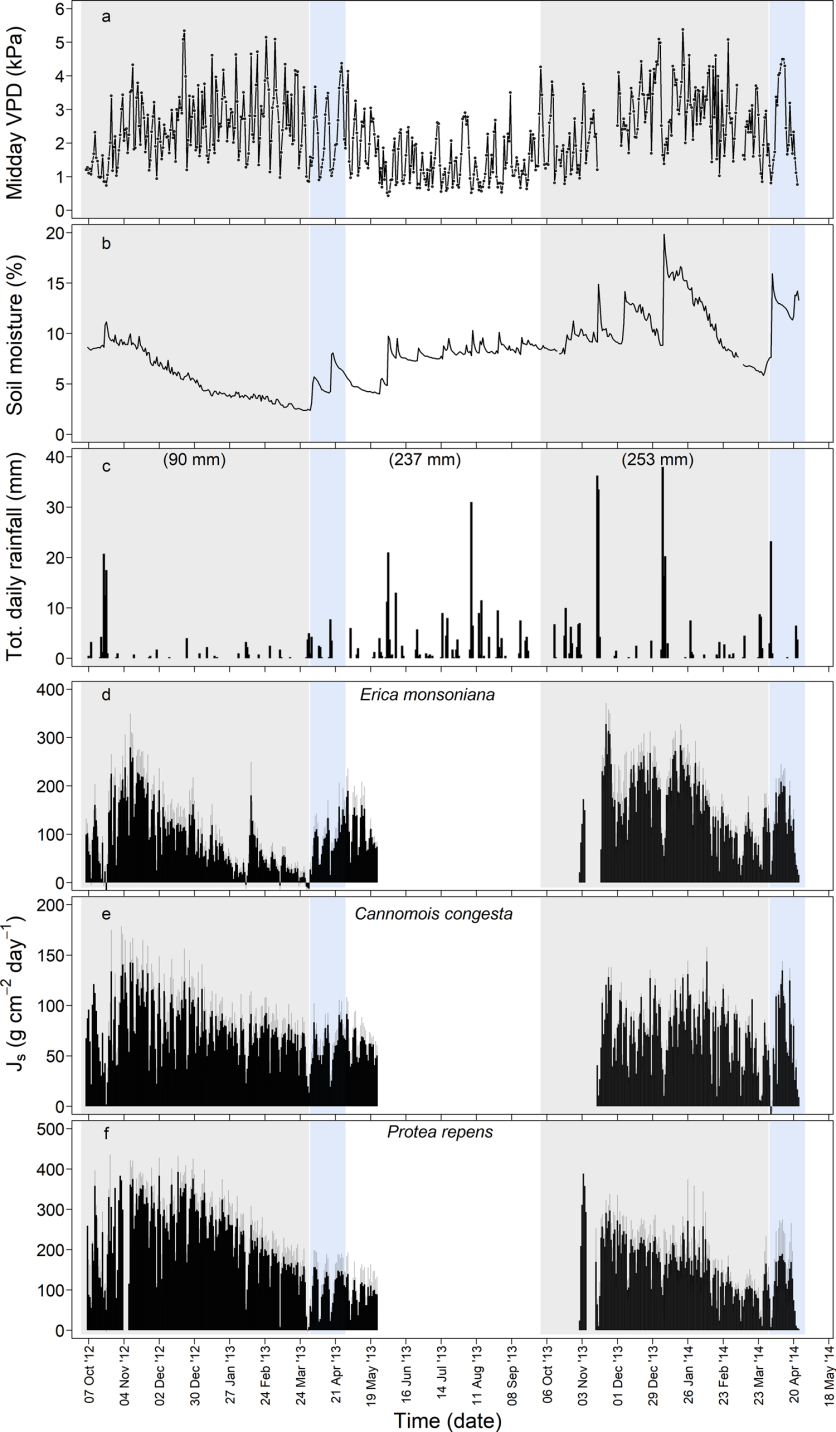
Timeline of environmental variables and total daily sap flux density (mean *J*_s_ ± SE) for each species recorded at the study site over the course of the study period. **a** Mean daily midday vapour pressure deficit (VPD, kPa); **b** soil moisture measured at a soil depth of 50 cm (%); **c** total daily rainfall (mm); and total daily sap flux density (g cm^−2^ day^−1^) for *Erica monsoniana* (**d**), *Cannomois congesta* (**e**) and *Protea repens* (**f**). Six-month total rainfall amounts (for summer 2012/2013, winter 2013, and summer 2013/2014) are indicated in parentheses. Grey background shading indicates summer periods, and blue shading indicates periods when soil moisture recovered following rehydrating rainfall events (see Methods for further details). Note the different *y*-axis scales in panels **d**–**f**

We captured sap flow across two summers, the first being drier than the second (Fig. 1). VPD patterns at the study site tended to be similar across years (e.g. highest during mid to late summer in both study years; Fig. 1a), and soil moisture started high in both summers (e.g. approximately 10% at 70 cm; Fig. 1b). Soil moisture declined steadily throughout the first summer (2012/2013; Fig. 1B), reaching the lowest levels observed in the study period in March 2013 (Fig. 1b). By comparison, soil moisture remained higher throughout 2013/2014, despite a consistent drying period between February and March 2014 (Fig. 1b).

Soil moisture patterns tended to be driven by summer rainfall inputs: total summer rainfall in 2012/2013 (90 mm) was less than half the amount received in 2013/2014 (253 mm) (Fig. 1c). Although several small rain events (< 10 mm) were recorded during both summers, three large (> 20 mm) rain events were recorded in the summer of 2013/2014 (Fig. 1c). Overall, the total annual rainfall in 2012 (293 mm) was also below (< 70%) the historical mean annual rainfall, while in 2013 the total amount (389 mm) was within 95% of the historical mean (Table S1). Dew and/or cloud moisture was routinely recorded at the site, with most events lasting more than 2 h (Fig. S2). Over the course of the study period, it was rare not to experience dew or cloud at the study site for longer than 4 days (Figure S2).

### Environmental drivers of within-season patterns of plant water use

Total daily sap flux density (*J*_s_) declined with the progression of summer in all three study species, although there were notable inter-specific differences in within- and between-season responses (Fig. 1d–f). In the drier year, total daily *J*_s_ of *Erica* declined earliest and to the lowest minimum (< 5% of maximum total daily *J*_s_) of the three species (Fig. 1d). *Protea*, which had the highest *J*_s_ in absolute terms, and *Cannomois*, which had the lowest *J*_s_, both maintained higher sap flow in the representative dry year compared to *Erica* (Fig. 1e, f). Nevertheless, total daily *J*_s_ of *Protea* declined steadily through the measurement period in 2012/2013, reaching as low as ~ 10% of maximum total daily *J*_s_. Total daily *J*_s_ of *Cannomois* was the least sensitive, remaining above 20% of maximum total daily *J*_s_ throughout the measurement period in 2012/2013. Total daily J_s_ remained high in the wetter year, remaining above ~ 20% of maximum in all three species. However, steep declines in total daily *J*_s_ were observed in *Erica* during dry spells, while more gradual declines were observed in *Protea* and *Cannomois* (Fig. 1d–f).

**Fig. 2:**
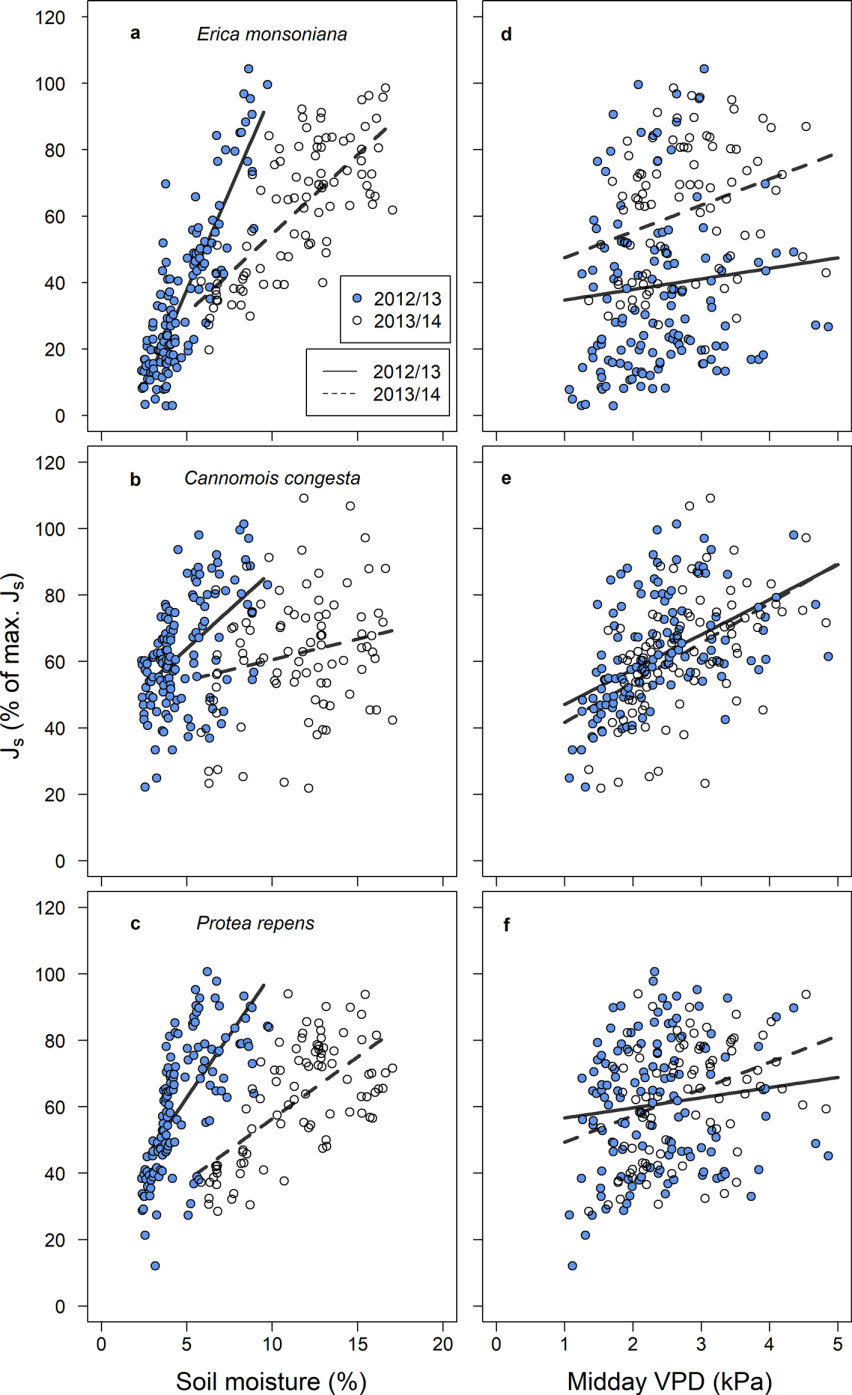
Relationship between total daily sap flux density (mean *J*_s_) and soil moisture or midday vapour pressure deficit (VPD) for the three study species in the representative dry year (2012/2013) and the normal year (2013/2014). Relationships between variables were assessed using linear mixed effects models

Total daily *J*_s_ was positively linearly associated with soil moisture in all three species (Tables S3, S4; Fig. 2a–c), with the steepest response being observed in *Erica* (Table S3; Fig. 2a). There were significant seasonal effects on the relationship between total daily *J*_s_ and soil moisture (Table S4), with the gradient of the response being steeper in all species in the representative dry season compared to the normal year (Table S3; Fig. 2a–c). A species by season interaction was also observed (Table S4), where there were weak positive associations between total daily *J*_s_ and soil moisture in the normal season in *Cannomois* and *Protea* (Table S3; Fig. 2b). Total daily *J*_s_ was also positively linearly associated with VPD in all species (Table S3, S4; Fig. 2d–f). Similar positive responses were recorded across seasons, although there was a significant species by season interaction (Tables S3, S4; Fig. 2d–f). We detected weaker positive associations between total daily *J*_s_ and VPD in the representative dry season than in the normal year for *Erica* and *Protea* (Table S3; Fig. 2d, f).

**Fig. 3:**
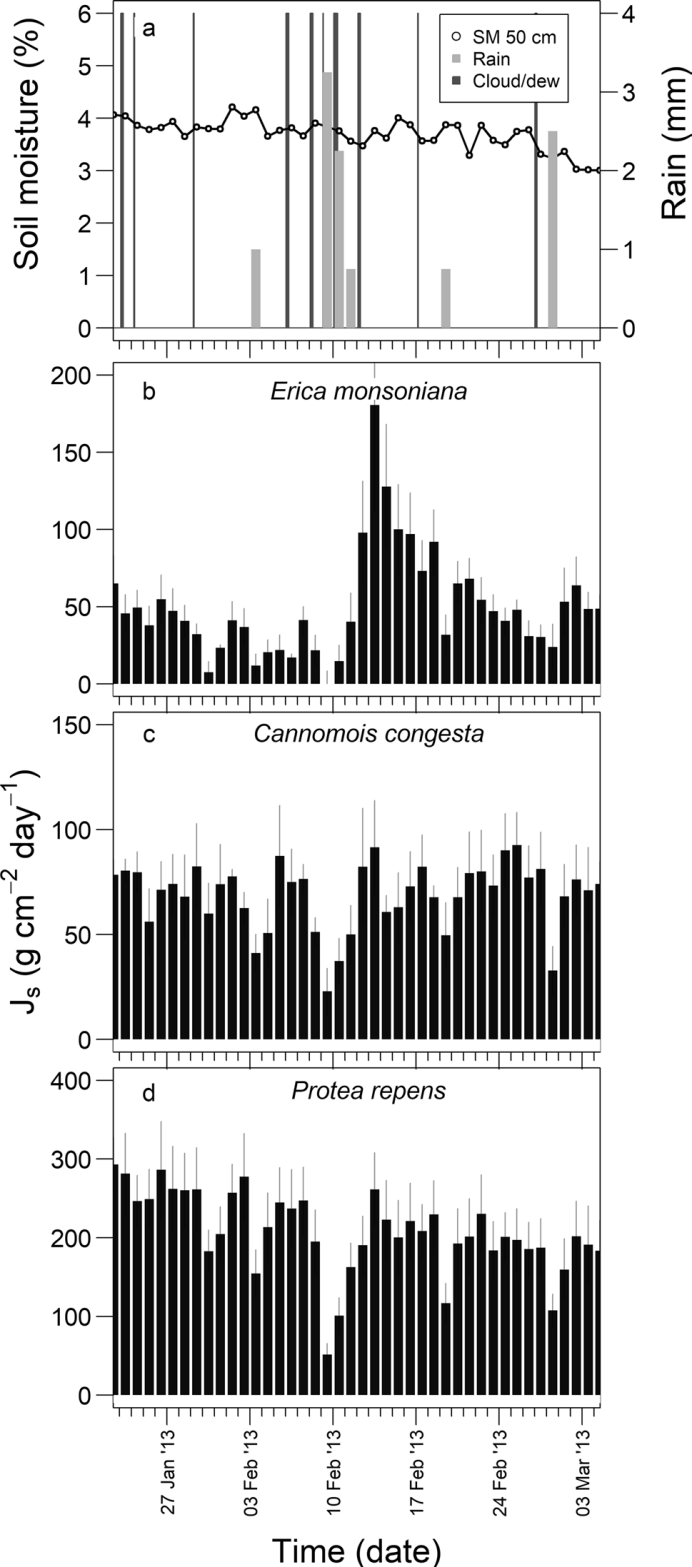
Timeline of environmental variables and total daily sap flux density (mean *J*_s_ ± SE) for each species recorded at the study site over mid-summer during the representative dry year (2013). **a** Mean daily soil moisture measured at a soil depth of 50 cm (%), total daily rainfall (mm), and duration of cloud/dew events (note that the thickness of the lines for each cloud/dew event is proportional to the duration of the event); **b**–**d** total daily sap flux density (g cm^−2^ day^−1^) for the three species

Total daily *J*_s_ was more responsive to summer rainfall event in *Erica* compared to the other two species. Figure 3 shows an example of summer rainfall that did not appear to reach the deeper soil layers (Fig. 3a) yet was associated with a substantial (> fivefold) increase in *J*_s_ in the few days following the event in *Erica* compared to the two weeks prior (Fig. 3b). The small rain inputs did not elicit substantial responses in the other two species (both < 1.4-fold increases) (Fig. 3c, d).

### Internal drivers of plant water use

**Fig. 4:**
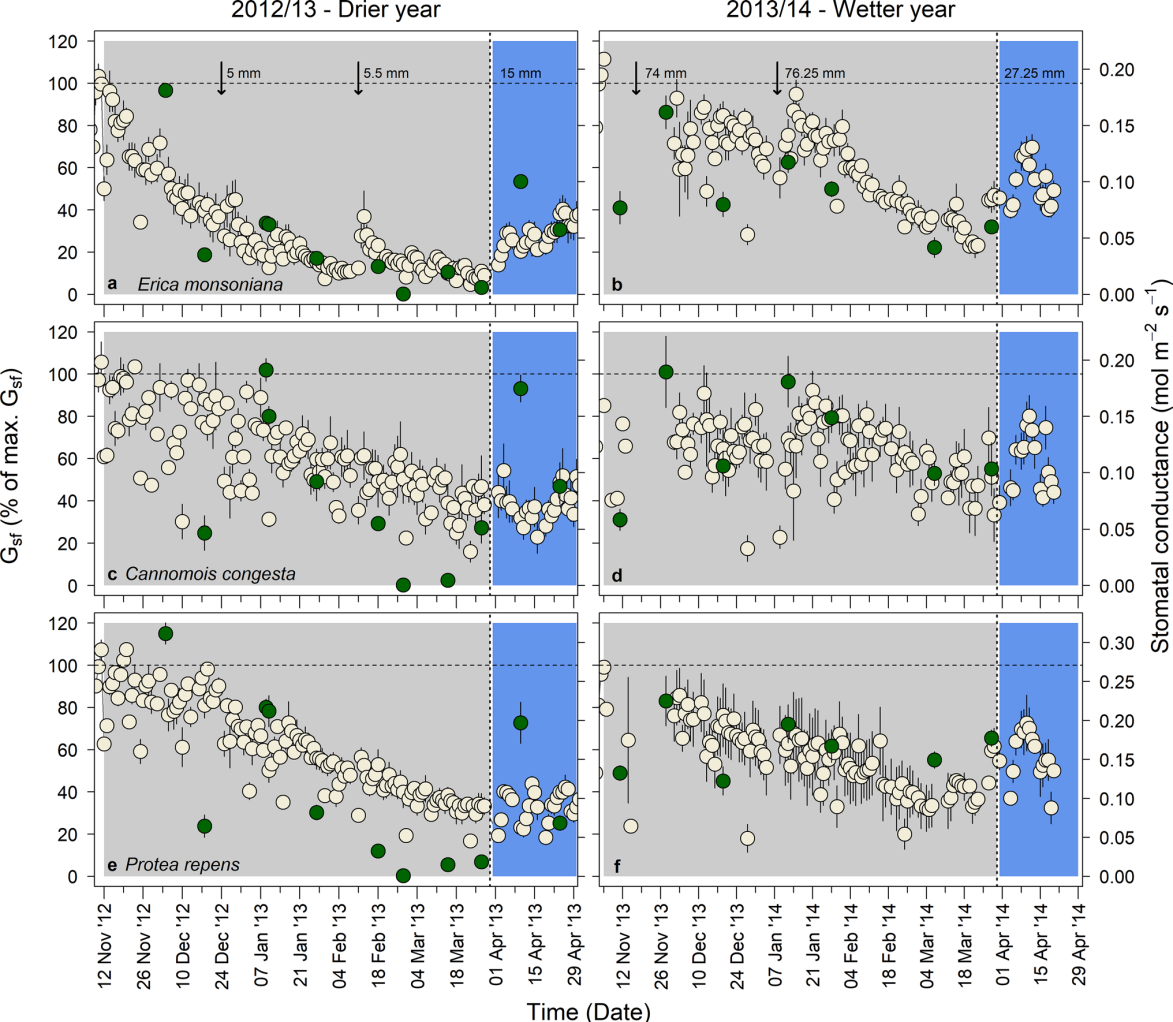
Timeline of mean midday sap flow-derived stomatal conductance (*G*_sf_; mean ± SE) expressed as a percentage of pre-stressed maximum *G*_sf_ for *Erica monsoniana* (**a**, **b**), *Cannomois congesta* (**c**, **d**), and *Protea repens* (**e**, **f**). Note: each row of panels is a timeline for the same species, but in two different years. Grey shaded areas indicate periods in summer when plant xylem water potentials generally declined, and blue shaded areas indicate periods when plant xylem water potentials recovered to pre-stressed values. Arrows indicate small summer rain events, and vertical dashed lines indicate large rainfall events. For comparison, the leaf level stomatal conductance is also shown (green points; mean *g*_s_ ± SE)

Seasonal patterns in sap flow-derived midday stomatal conductance (*G*_sf_) were similar to the overall patterns in total daily *J*_s_ in all three species (Fig. 4). In the drier year, *G*_sf_ of *Erica* declined earliest and to the lowest minimum of the three species (Fig. 4a). *G*_sf_ of *Erica* fell below 30% in late December 2012 and thereafter to a minimum of < 5% in March 2013 (Fig. 4a). In comparison, *Protea* and *Cannomois* both maintained higher *G*_sf_ for longer in the representative dry year (Fig. 4b, c). *G*_sf_ of *Protea* declined to c. 30% in March 2013, approximately 84 days after *Erica* hit the same level. *G*_sf_ of *Cannomois* was the least sensitive out of the three study species, falling to c. 30% of maximum G_sf_ in early April 2013, about 100 days after *Erica* had reached this value. *G*_sf_ remained high in the normal year, declining to ~ 20% of maximum in *Erica*, but remaining above 30% in *Cannomois* and *Protea*.

Changes in midday stomatal conductance (*g*_s_) measured at the leaf or culm level typically matched those observed at the shoot level (*G*_sf_) in all three sample species (Fig. 4). Minor differences in leaf/culm versus shoot values arose during periods of very low moisture availability or immediately following rehydrating rainfall events. Such minor discrepancies likely reflect the difference between an integrated sap flow-derived measurement taking into account the function of all leaves on a given shoot versus that of a healthy, sunlit leaf/culm. For example, lower leaf or culm g_s_ during drier periods could reflect midday stomatal closure in sunlit leaves versus integrated sap flow of sunlit and shaded leaves. Higher leaf or culm g_s_ following rehydration could reflect the function of a healthy sunlit leaf versus integrated function of damaged and healthy leaves (Fig. 4).

### Recovery of stomatal conductance and water use following rehydration

**Fig. 5:**
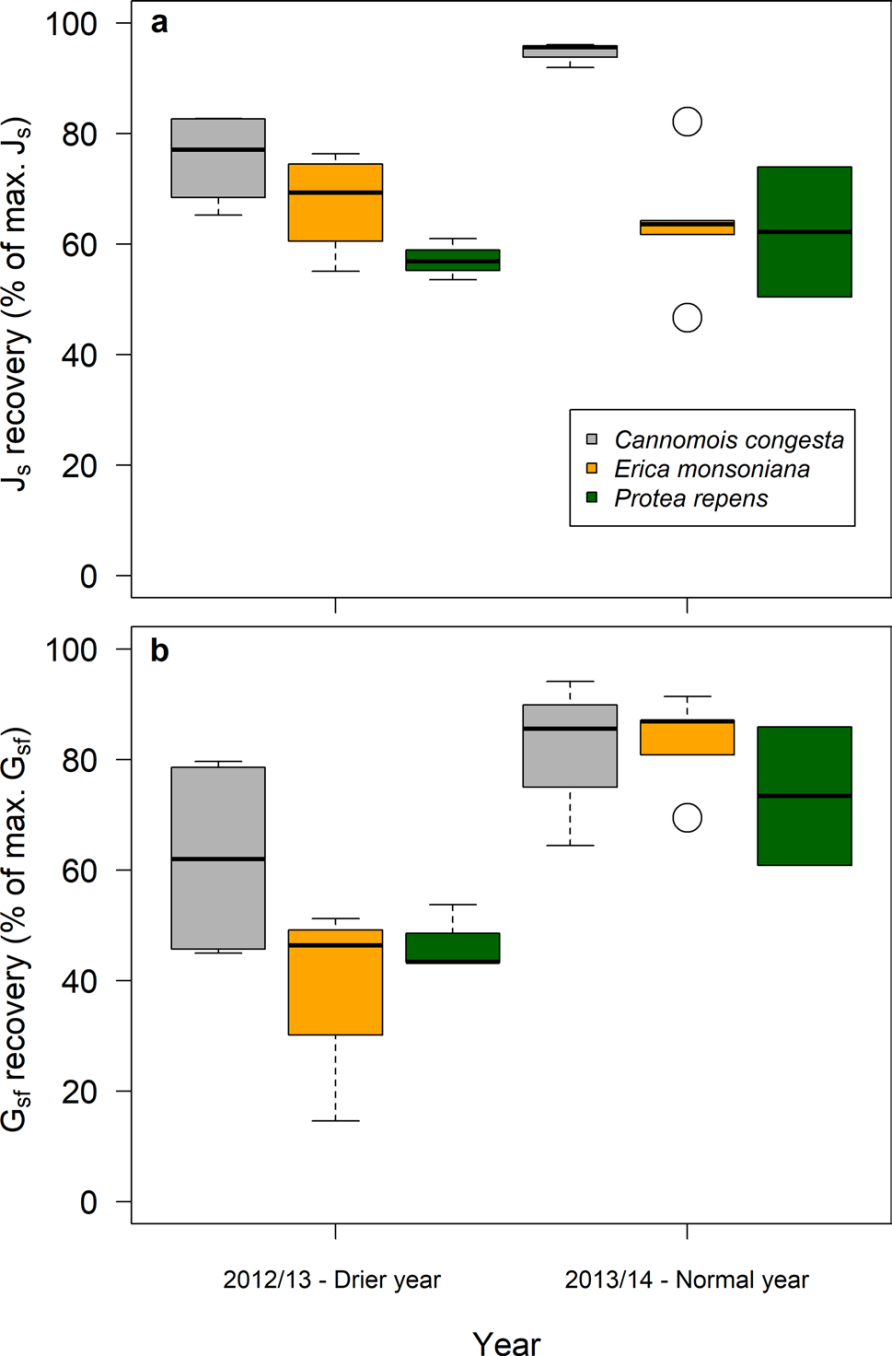
The recovery of total daily *J*_s_ (**a**) and *G*_sf_ (**b**) for each species in the representative dry year (2012/2013) and the normal year (2013/2014). Horizontal bars represent the minimum and maximum (outer bars connected by dashed lines), the median (inner, thick lines), and the first quartile and third quartile in the dataset of each species in the two seasons (coloured sections). Open circles represent outliers

Following large rainfall events (> 15 mm) total daily J_s_ rose in all three species, but the extent of the recovery differed between species (Table 1). Recovery was higher in *Cannomois* compared to *Erica* (*p* = 0.01) and *Protea* (*p* < 0.002), but did not differ between the latter two species (*p* = 0.38) nor between seasons (Table S5). In the drier season, total daily *J*_s_ rose to c. 67% of pre-stressed total daily J_s_ in *Erica*, similar to the recovery recorded for a similar event in the second, normal season (Fig. 5a). In *Cannomois*, total daily *J*_s_ recovered to > 75% of pre-stressed total daily *J*_sf_ in the first season and to 95% in the second season (Fig. 5a). In both seasons recovery of total daily *J*_s_ was lowest in *Protea*, only reaching 57% of pre-stressed total daily *J*_s_ in the drier year and 62% in the normal season (Fig. 5a).

**Table 1:** ANOVA table showing main effects results of the linear model of recovery of total daily J_s_ (% of maximum J_s_) or G_sf_ (% of maximum G_sf_) as a function of species and/or season

Response variable	Explanatory variables	*D.F*	Sum Sq	*F*	*P*
Total daily *J*_s_	Species	**2**	**2338**	**9.7**	**0.001**
	Residuals	18	2169		
G_sf_	Season	**1**	**4998**	**22.2**	**0.0001**
	Residuals	19	42,710		

The two models presented here performed better than the full model, which included both species and season as well as an interaction between them (see Methods and Supplementary material for details). Statistically significant effects are indicated in bold

Recovery patterns of *G*_sf_ following large rainfall events differed from those for total daily *J*_s_, in that recovery of *G*_sf_ was lower in the representative dry year in comparison to the normal year (Table 1) and did not differ between species (Table S5). In the drier season, *G*_sf_ rose to c. 40% of pre-stressed *G*_sf_ in *Erica*, half the recovery recorded for a similar event in the second, normal season (Fig. 5b). In *Cannomois* G_sf_ recovered to 62% of pre-stressed *G*_sf_, less than the 81% recovery recorded in the second season (Fig. 5b). *G*_sf_ was lower in the drier year in *Protea*, recovering to c. 47% of pre-stressed *G*_sf_, less than the 73% recovery recorded in the second, normal season (Fig. 5b).

### In situ changes in leaf or culm water potential

**Fig. 6:**
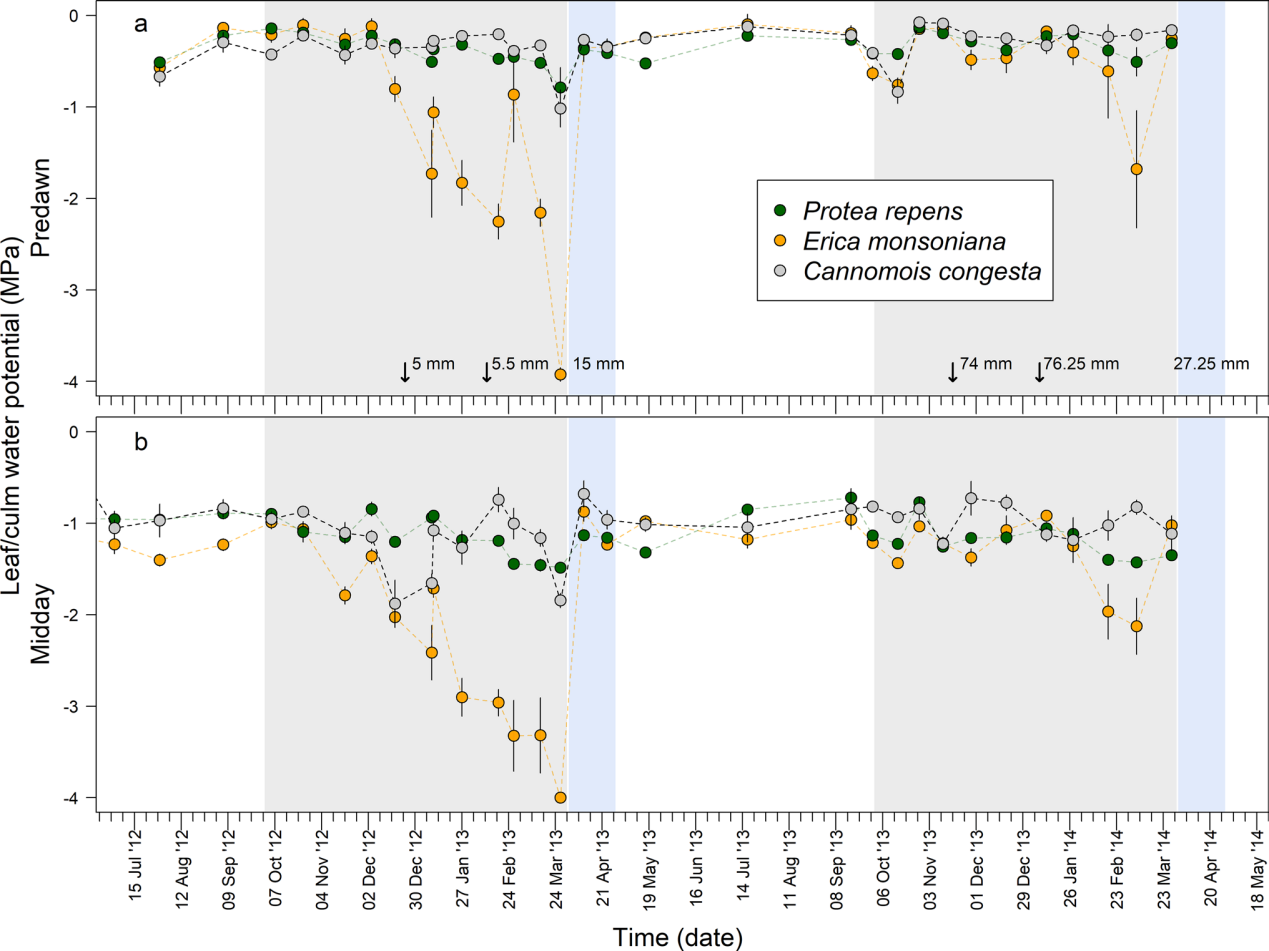
Timeline of predawn leaf or culm water potential (**a**) and midday leaf or culm water potential (**b**) (mean ± SE, *n* = 5) for the three study species over the course of the two summer study periods. Grey background shading indicates summer periods, and blue shading indicates periods when soil moisture recovered following rehydrating rainfall events

Minimum predawn and minimum midday leaf or culm water potentials differed between species and seasons (Fig. 6; Tables 2, S6). Predawn leaf or culm water potentials remained high (i.e. less negative than − 1 MPa) and stable in *Protea* and *Cannomois*, but were more dynamic and declined lower (< − 1.5 MPa) in *Erica* (Fig. 6a). Seasonal patterns of midday leaf or culm water potentials were similar to those observed for predawn leaf or culm water potential (Fig. 6b). Minimum midday values were more negative in *Erica* (− 4.0 ± 0.1 MPa in March 2013) than in *Protea* (− 1.5 ± 0.03 MPa, *p* < 0.000) and *Cannomois* (− 1.9 ± 0.26 MPa, *p* < 0.000), but did not differ between the latter two species (*p* = 0.94) (Fig. 6 and Table 2). Although minimum midday leaf or culm water potentials tended to be lower in the drier summer of 2012/13 (*p* < 0.000), there was a significant species by season interaction (Table S6). Post hoc tests revealed that only *Erica* was significantly more dehydrated in 2012/2013 than in 2013/2014 (Table 2).

**Table 2:** A brief description of each study species’ growth form together with key physiological measurements, including the minimum leaf or culm xylem water potential (mean ± SE) recorded for each species at the study site during two seasons, the species stem or culm P_50_ (mean ± SE), and the predicted percentage loss of xylem function in each study season based on xylem vulnerability curves and the minimum leaf or culm water potential. Numbers in round brackets indicate sample sizes for minimum leaf or culm water potential and xylem vulnerability curves

Species	Growth form	Minimum leaf or culm water potential (MPa)		P_50_ (MPa)	Predicted loss of xylem function (%)	
		2012/2013	2013/2014		2012/2013	2013/2014
*Erica monsoniana*	Ericoid; Medium-sized, shallow rooted, small-leaved, woody shrub	− 4.0 ± 0.1 (5)^a^	− 2.13 ± 0.31 (5)^b^	− 5.68 ± 0.78 (3)^b^	7.3	0.5
*Protea repens*	Proteoid; Large, deep rooted, broad-leaved, woody, overstorey shrub	− 1.5 ± 0.03 (5)^b,c^	− 1.42 ± 0.03 (5)^b,c^	− 3.50 ± 0.06 (4)^a^	0	0
*Cannomois congesta*	Restioid; shallow-rooted, rhizomatous perennial with erect photosynthetic culms	− 1.9 ± 0.26 (5)^b,c^	− 1.18 ± 0.25 (5)^c^	− 2.30 ± 0.12 (3)^a^	1.8	0

Letters indicate results of Tukey multiple comparisons of means test for the comparisons between species for each measurement

**Fig. 7:**
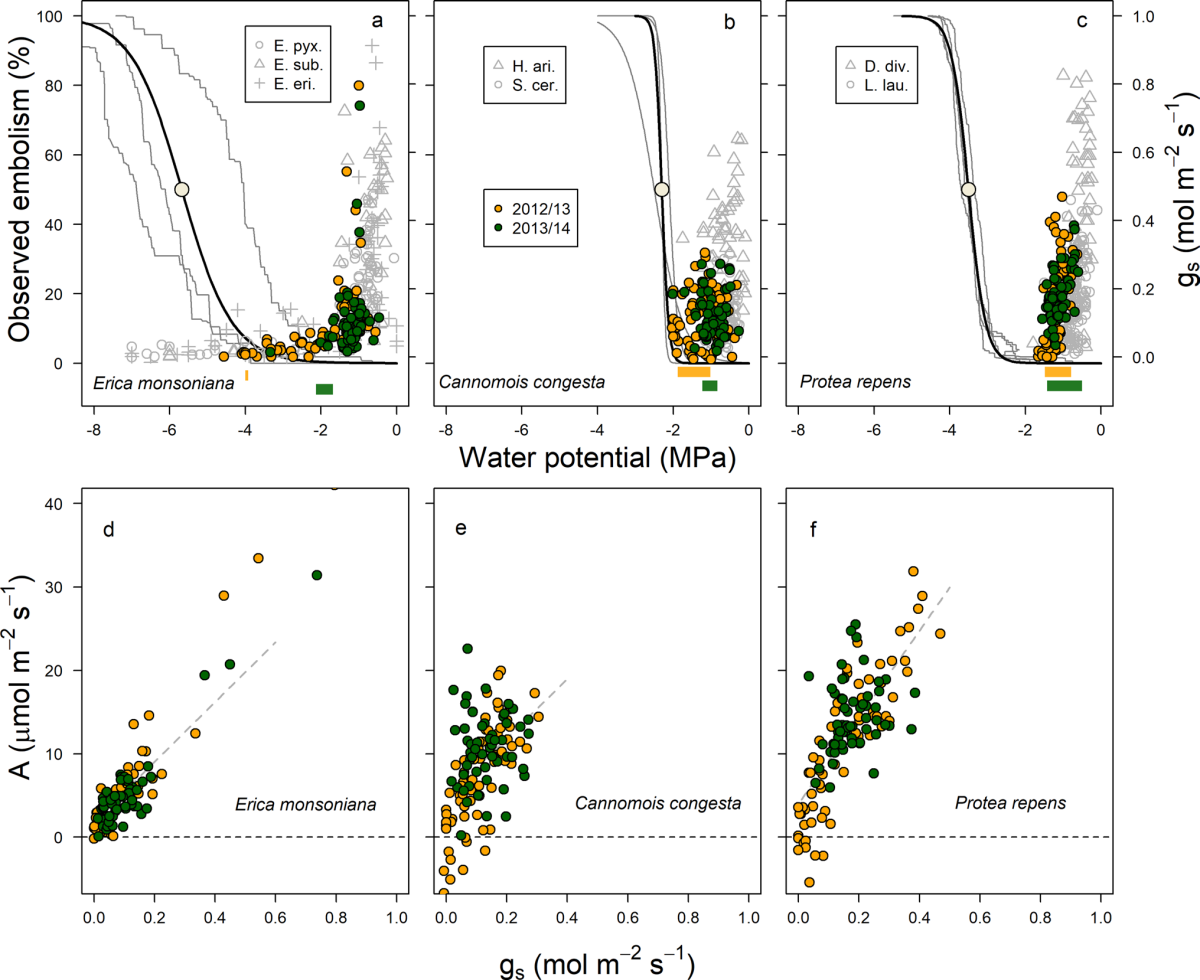
**a**–**c** Stomatal conductance, water potential envelopes for the three Jonaskop species (orange and green points are from the same species in two different years) and for Silvermine species from West et al. 2012 (grey points). The Silvermine species are *Erica ericoides*, *Erica pyxidiflora* and *Erica subcapitata* (in panel a); *Hypodiscus aristatus* and *Staberoha cernua* (in **b**); and *Diastella divaricata* and *Leucadendron laureolum* (in **c**). Also shown are the xylem vulnerability curves for the three Jonaskop species: solid black lines are mean xylem vulnerability curves, grey lines are curves for individual plants, and filled white circles indicate the P_50_ values for each species. Minimum xylem water potentials for each species are also shown. Bars indicate the range between minimum predawn and minimum midday water potentials recorded in 2012/2013 and 2013/2014. **d**–**f** The relationship between stomatal conductance and carbon assimilation measured for all three Jonaskop species in the representative dry year (2012/2013, orange) and the normal year (2013/2014, green). Grey lines are best fit models of the relationships

Leaf or culm water potentials of all three species occasionally recovered to less negative values throughout both summers (Fig. 6). Partial recovery of leaf or culm water potential was associated with small moisture inputs (< 10 mm; see arrows in Fig. 6). All three species rehydrated to water potentials matching those of unstressed conditions following large (> 15 mm) summer rainfall events (blue shaded areas in Fig. 6). In all species, stomatal conductance declined rapidly with declining leaf or culm water potential, with stomata being mostly closed at water potentials above (i.e. less negative than) − 2 MPa (Fig. 7).

### Predicted loss of function in the stem/culm xylem transport system

Stem or culm xylem capacity to withstand embolism differed between the three study species (Fig. 7; *F* = 16.36, *d.f.* = 2, *p* = 0.0023). The water potential associated with 50% embolism varied from − 2.3 ± 0.12 MPa in culms of *Cannomois* to − 5.68 ± 0.78 MPa in stems of *Erica* (Table 2). When combined with the seasonal minimum leaf or culm water potential, the vulnerability curves indicate that *Cannomois* and *Erica* were likely to have surpassed thresholds associated with embolism formation in culms/stems in 2012/2013, but not in 2013/2014 (Fig. 7; Table 2). The predicted amount of embolism to have occurred in 2012/2013 (i.e. from the combination of the species' mean stem/culm vulnerability curves and the minimum water potentials from that season) was < 10% in both species (Fig. 7; Table 2). When combined with the seasonal minimum leaf or culm water potential, the vulnerability curves indicate that *Protea* was unlikely to have experienced embolism in the stem xylem in either 2012/2013 or 2013/2014 (Fig. 7; Table 2).

## Discussion

Our novel, multi-year sap flow dataset, collected on multiple growth forms simultaneously, together with associated physiological measurements, provides unique insight into the ecology of the CFR. Our study is consistent with previous studies, providing strong confirmatory evidence of consistent functional responses within mountain fynbos, adding weight to the functional classifications inferred from snapshot campaigns conducted on distinct species at different field sites. We also report on the novel finding that despite co-occurring species with different growth forms experiencing quite different responses to dehydration, these did not translate into substantial variation in recovery following rehydration, primarily due to differences in xylem vulnerability to embolism and water uptake characteristics.

### Consistent functional responses within closely related species with similar growth form

Our highly temporally resolved dataset revealed functional responses to the environment in our three species that were consistent with previous studies conducted on congenerics at separate locations and at separate times. *Erica monsoniana* was most tightly coupled to shallow moisture dynamics through the summer, showing rapid declines in sap flow and gas exchange as the surface soils dried. This was associated with large declines in xylem pressure potentials over the summer, an observation consistent with previous studies (Miller et al. 1983, 1984; West et al. 2012). The declines in water potential were greater in the drier year, and may have resulted in xylem embolism in *Erica* (Fig. 7), highlighting the importance of summer moisture for this species. This is further supported by the clear uptake of summer rainfall pulses by *Erica* (Fig. 3) indicating the presence of active roots in shallow soil layers during the summer. This conclusion is consistent with observations that *Erica* species typically have shallow roots (e.g. *Erica plukenetii* Higgins et al. 1987). Our observations also suggest that prior observations showing a lack of response to irrigation in other mountain fynbos species (e.g. van der Heyden and Lewis 1990) may be incomplete, possibly because these plants were sampled in a wetter year than in our study. For example, van der Heyden and Lewis (1990) showed that *Erica plukenetii* did not increase its photosynthetic rates when irrigated concluding that it did not use the additional water as soon as it became available in the soil and that it was not limited by low soil water availability during February. In contrast, our results show that plant gas exchange capacity in *Erica monsoniana* was sensitive to summer rainfall, declining steeply during drying periods and responding rapidly to small summer rainfall events (in the drier year). Such dynamic stomatal responses caused by rapidly changing leaf water potentials suggest that not only does *Erica monsoniana* have active roots in shallow soil layers over the summer, but that it may also exhibit a peaking-type ABA response previously documented in conifers (Brodribb et al. 2014).

In contrast, patterns of leaf water potential and sap flow decline (and recovery) in *Protea repens* were better explained by variation in soil moisture of deeper layers, consistent with previous studies that have found proteoid species are deeper-rooted (e.g. *Protea laurifolia* in Higgins et al. 1987; *Leucadendron laureolum* in West et al. 2012). Unlike *Erica*, *Protea* did not respond to small summer rainfall events, suggesting that *Protea repens* does not have active roots in shallow soil layers over the summer. *Protea repens* also maintained high leaf water potentials throughout both summer periods, with only gradual declines to more negative leaf water potentials through drier periods. Our sap flow and gas exchange data showed that this maintenance of high water potentials was not solely a function of having access to deeper soil moisture but was in part due to stomatal closure during the summer. This observation is consistent with the responses seen in the proteoid *Leucadendron laureolum* (West et al 2012), suggesting that maintenance of high water potentials is important for the success of this functional type. A plausible reason is that they are weakly serotinous species and reductions in leaf water potentials may trigger premature (i.e. not post-fire) seed release with consequent reductions in reproductive fitness. However, having stomata closed during late summer when temperatures are still high reduces the capacity for transpirational leaf cooling and may induce thermal stress, particularly under windless days (Herppich et al. 1994; Yates et al. 2010, Karpul and West 2016). Thermal stress, if it occurs, is most likely to impact the broad-leafed *Protea* and not *Cannomois* or *Erica*. *Cannomois* has narrow, vertical culms, while *Erica* has small, rolled leaves, which may promote efficient sensible heat loss, thereby reducing the requirement for transpirational leaf cooling in summer.

Excavations done at the study site showed that the restioid, *Cannomois,* had dense, shallow (< 40 cm) adventitious root systems (Fig. S3), consistent with in situ observations made on other Restionaceae species (e.g. *Ischyrolepis* and *Elegia*, Higgins et al. 1987, *Hypodiscus* and *Staberoha*, West et al. 2012). Furthermore, this species maintained high water potentials throughout the summer periods and had the lowest inter-annual reduction in G_sf_ among the study species. This too is consistent with previous observations of water potentials in mountain fynbos Restionaceae subjected to drought (West et al 2012). Our explanation for the observation that *Cannomois* remains hydrated even during periods with low rainfall is that these individuals maintain low rates of water use (in absolute terms) by utilizing dew or cloud moisture inputs in addition to the soil moisture. Dew events lasting over two hours were common at the study site (Fig. S2). Previous research in other species of Restionaceae in mountain fynbos communities (Marloth 1903, 1905; Nagel 1956) has shown that culms can trap considerable amounts of moisture from the atmosphere, decoupling the plant from surface soil moisture during drought (West et al 2012), maintaining plant function in periods without substantial rainfall. Maintenance of high water potentials through the end of summer may be particularly important for nut-fruited species like *Cannomois* that take 2 years to mature seeds in their canopy. Culms that desiccate below ±  − 2.5 MPa tend to drop their seeds prematurely (pers. obs.). Establishing how long *Cannomois* (and other Restionaceae species) can survive without dew/cloud moisture is a research priority for understanding potential drought impacts in the CFR.

Overall, our three sample species displayed physiological responses to seasonal water limitation that are consistent with the ericoid, proteoid and restioid functional type responses characterised by West et al. (2012) under more severe experimental drought conditions. This is most noticeable in the similar stomatal and leaf or culm water potential responses observed for our species and for those from the same growth forms but occurring in a different mountain fynbos community located > 100 km away from our study site. (i.e. compare different species within each panel in Fig. 7a–c). Further, the reduced g_s_ seen in all three species during two prolonged dry periods were consistent with prior observations of seasonal declines in g_s_ for mountain fynbos species, including in congeneric species to our study species (e.g. *Erica plukenetii*, *Protea laurifolia* and *Cannomois acuminata* in Miller et al. 1983). This lower stomatal conductance was associated with lower photosynthesis at the leaf level (Fig. 7d–f), indicating that dry summer spells reduce the overall productivity of mountain fynbos communities.

### Convergence in recovery following rehydration despite differing minimum water potentials and hydraulic safety margins

By comparing the within-season recovery of *G*_sf_ of a normal year with that of a representative dry year, we were able to determine that gas exchange across all sample species was limited by up to 30% in the representative dry year. The species converged in their recovery of *G*_sf_, despite the substantial variation in minimum water potentials, due to the significant variation in xylem vulnerability to embolism between our study species that matched the order of seasonal minimum leaf or culm water potentials (i.e. *Erica* <  < *Protea* < *Cannomois*). This resulted in all three species maintaining positive hydraulic safety margins from P_50_, albeit to differing degrees. Both shallow-rooted species (i.e. *Erica* and *Cannomois*) exhibited small xylem hydraulic safety margins, and both were predicted to have experienced a slight loss of function due to embolism in the driest year. In contrast, *Protea* maintained a much larger stem hydraulic safety margin, indicating that of the three species it is the least likely to incur hydraulic failure in stems. Co-variation of physiological responses and functional traits suggests an evolved response to seasonal drying in mountain fynbos communities that resulted in convergent recovery of differing functional types during two “normal” years (i.e. not severe drought). This is unsurprising, given that these species have evolved to exist in this climate. However, it is less certain whether this convergence would continue should these communities be exposed to extreme drought induced by climate change.

### The potential impacts of future droughts on mountain fynbos communities

The results of this study under moderate drying conditions are consistent with previous experimental drought studies conducted in the CFR in which plants were subject to more extreme drought. For example, after experimentally removing summer rainfall in their field manipulation study, West et al. (2012) showed *Erica* species had reduced growth and flowering output and the highest levels of dieback and mortality, while restioid and overstorey proteoid species displayed little treatment effect on growth, flowering output and mortality. This is well aligned with our finding of the vital role that small, but regular summer rainfall events play in the maintenance of function in *Erica monsoniana*. Another experimental drought study conducted on seedlings of several Proteaceae species demonstrated that seedlings possess an ability to persist for prolonged periods without watering (Arnolds et al. 2015), suggesting that West et al.’s observed responses apply across life history stages in this functional type. Our study suggests that knowledge of the quantitative physiological responses of a few species representing dominant functional types can be used to make meaningful predictions about future drought impacts in hyper-diverse plant communities such as the mountain fynbos. We do, however, note that West et al. demonstrated some variability of responses within families that was related to variable growth form (e.g. within the Proteaceae) and it remains to be determined whether congenerics with dissimilar growth forms exhibit similar physiological strategies in spite of large morphological differences.

## Conclusion

Co-occurring fynbos species rely on subtly different water sources, which may confer differential sensitivity to changing climate. The disproportionate role that infrequent but substantial summer rainfall events play in maintaining gas exchange capacity in *Erica* suggests that future reductions in rainfall amounts—or changes in rainfall seasonality that are associated with less summer rainfall—are most likely to negatively impact growth in this species. Further, *Erica*, but not *Protea* and *Cannomois*, had significantly smaller hydraulic safety margins in the representative dry year than in the normal year, suggesting that it is the most likely to experience hydraulic dysfunction and loss of function under extreme drought conditions. Overall, the three sample species displayed physiological responses to seasonal resource limitation that are highly consistent with the ericoid, proteoid and restioid functional type responses characterised by prior studies, suggesting that simplifying the extra-ordinary species diversity of the region into these key functional types provides a useful avenue for understanding functional responses to change in mountain fynbos.

## Supplementary Information

Below is the link to the electronic supplementary material.

Supplementary file1 (DOCX 2149 KB)

## Data Availability

The datasets used and/or analysed during the current study are available from the corresponding author on reasonable request.

## Code availability

The R code scripts used during the current study are available from the corresponding author on reasonable request.
