# Supplementary material for: Consistent responses to moisture stress despite diverse growth forms within mountain fynbos communities
Source: Oecologia. 2023 Jan 24;201(2):323–39. doi: 10.1007/s00442-023-05326-9 (PMC9944370; doi:10.1007/s00442-023-05326-9)
Supplement: Supplementary file 1 — Supplementary file1 (DOCX 2149 KB) [file 442_2023_5326_MOESM1_ESM.docx]

## Supplementary Tables with Captions

**Table S1:** Comparison of historical rainfall averages (2003-2008) with rainfall measured during this study. Values in parentheses are percentages of the historical annual amount.

| **Time period** | **Rainfall amount (mm)** | | |
| --- | --- | --- | --- |
|  | **Winter^1^** | **Summer^1^** | **Annual^1^** |
| Historical |  |  | 411 |
| 2012 | 176 | 90 | 293 (71 %) |
| 2013 | 237 | 253 | 389 (95 %) |

1 Annual total is the total rainfall amount received in a calendar year (i.e., 1 January to 31 December). Winter represents total rainfall for the months from 1 April to 30 September; Summer represents total rainfall for the months from 1 October to 31 March the following year. Therefore, annual values do not equal the sum of the summer and winter values.

**Table S2:** The relationship between *in situ* midday transpiration of sunlit leaves/culms measured with a portable open gas exchange system (Li-Cor 6400, Li-Cor Biosciences) and sap flow of sunlit leaves/culms measured with miniature external sap flow gauges was strong and highly significant for individuals of all three sample species at the study site.

| **Species** | **Season** | **Individual** | **slope** | **r^2^** | **p** | **F** | **d.f.** |
| --- | --- | --- | --- | --- | --- | --- | --- |
| ***Erica monsoniana*** | **2012/13** | **1** | 0.13 | 0.73 | 0.028 | 26.67 | 10 |
|  |  | **2** | 0.09 | 0.71 | 0.0235 | 24.68 | 10 |
|  |  | **3** | 0.11 | 0.69 | 0.0305 | 21.76 | 10 |
|  |  | **4** | 0.24 | 0.78 | 0.0007 | 28.2 | 8 |
|  |  | **5** | 0.26 | 0.92 | 0.0000 | 99.21 | 9 |
|  | **2013/14** | **1** | 0.15 | 0.97 | 0.0004 | 113.46 | 4 |
|  |  | **2** | 0.23 | 0.95 | 0.0009 | 76.68 | 4 |
|  |  | **3** | 0.17 | 0.91 | 0.0034 | 38.96 | 4 |
|  |  | **4** | 0.3 | 0.97 | 0.0025 | 90.18 | 3 |
|  |  | **5** | 0.24 | 0.72 | 0.0019 | 20.44 | 8 |
|  |  | **6** | 0.22 | 0.99 | 0.0000 | 359.96 | 5 |
| ***Cannomois congesta*** | **2012/13** | **1** | 0.15 | 0.64 | 0.0018 | 17.72 | 10 |
|  |  | **2** | 0.12 | 0.57 | 0.0045 | 13.32 | 10 |
|  | **2013/14** | **1** | 0.15 | 0.98 | 0.0000 | 250.69 | 5 |
|  |  | **2** | 0.13 | 0.83 | 0.0003 | 38.53 | 8 |
| ***Protea repens*** | **2012/13** | **1** | 0.15 | 0.3 | 0.0529 | 4.7 | 11 |
|  |  | **2** | 0.16 | 0.81 | 0.0001 | 43.72 | 10 |
|  |  | **3** | 0.23 | 0.73 | 0.0009 | 23.9 | 9 |
|  |  | **4** | 0.22 | 0.54 | 0.0104 | 10.42 | 9 |
|  |  | **5** | 0.17 | 0.63 | 0.0034 | 15.57 | 9 |
|  | **2013/14** | **1** | 0.09 | 0.84 | 0.0014 | 31.31 | 6 |
|  |  | **2** | 0.15 | 0.97 | 0.0001 | 155.27 | 5 |
|  |  | **3** | 0.12 | 0.93 | 0.0004 | 70.5 | 5 |

**Table S3:** Fixed effects results for the best fit linear mixed effects model of J_s_ as a function of soil moisture or VPD, species and season (fixed factors) and individual (random factor). *p<0.05; **p<0.01; ***p<0.001.

| **Best Fit Model** | **Explanatory variables** | **Estimate (± s.e.)** |
| --- | --- | --- |
| J_s_ ~ soil moisture * species * season + (1\|species/season/individual) | Int., S1: *Cannomois* | 40.5*** (3.4) |
|  | Slope, S1: *Cannomois* | 4.7*** (0.5) |
|  | Int., S1: *Erica* | −62.0*** (4.6) |
|  | Slope, S1: *Erica* | 7.2*** (0.6) |
|  | Int., S1: *Protea* | −15.2** (4.4) |
|  | Slope, S1: *Protea* | 2.8*** (0.6) |
|  | Int., S2: *Cannomois* | 7.6 (6.3) |
|  | Slope, S2: *Cannomois* | −3.4*** (0.6) |
|  | Int., S2: *Erica* | 20.8** (7.9) |
|  | Slope, S2: *Erica* | −3.7*** (0.8) |
|  | Int., S2: *Protea* | −13.8 (8.4) |
|  | Slope, S2: *Protea* | −0.3 (0.8) |
| J_s_ ~ VPD * species * season + (1\|species/season/individual) | Int., S1: *Cannomois* | 36.6*** (4.2) |
|  | Slope, S1: *Cannomois* | 10.5*** (1.2) |
|  | Int., S1: *Erica* | −5.0 (5.7) |
|  | Slope, S1: *Erica* | −7.3*** (1.7) |
|  | Int., S1: *Protea* | −17.0** (5.6) |
|  | Slope, S1: *Protea* | −7.5*** (1.7) |
|  | Int., S2: *Cannomois* | −6.9 (7.4) |
|  | Slope, S2: *Cannomois* | 1.4 (2.2) |
|  | Int., S2: *Erica* | 14.9 (9.3) |
|  | Slope, S2: *Erica* | 3.3 (2.8) |
|  | Int., S2: *Protea* | −5.4 (9.8) |
|  | Slope, S2: *Protea* | 3.5 (2.9) |

**Table S4:** Summary statistics for the linear mixed effects models fitted to the total daily J_s_ observed as a function of soil moisture or VPD, species and season including a three way interaction.

| **Response Variable** | **Fixed Effects** | **numDF** | **denDF** | **F-value** | **p-value** |
| --- | --- | --- | --- | --- | --- |
| **Total daily J_s_** | **soil moisture** | **1** | **2777** | **1357** | **< 0.0001** |
|  | **species** | **2** | **152** | **91** | **< 0.0001** |
|  | **season** | **1** | **153** | **10** | **0.002** |
|  | **soil moisture: species** | **2** | **2777** | **99** | **< 0.0001** |
|  | **soil moisture: season** | **1** | **2777** | **243** | **< 0.0001** |
|  | **species: season** | **2** | **152** | **12** | **< 0.0001** |
|  | **soil moisture: species: season** | **2** | **2777** | **16** | **< 0.0001** |
| ***Note:*** Full model: lmer(J_s_ ~ soil moisture*species*season + (1\|species/season/individual)) | | | | | |
| **Total daily J_s_** | **VPD** | **1** | **2756** | **174** | **< 0.0001** |
|  | **species** | **2** | **166** | **5** | **0.005** |
|  | season | 1 | 167 | 1 | 0.33 |
|  | **VPD: species** | **2** | **2756** | **10** | **< 0.0001** |
|  | **VPD: season** | **1** | **2756** | **10** | **0.001** |
|  | **species: season** | **2** | **166** | **3** | **0.05** |
|  | VPD: species: season | 2 | 2756 | 1 | 0.41 |
|  |  | | | | |
| ***Note:*** Full model: lmer(J_s_ ~ VPD*species*season + (1\|species/season/individual)) | | | | | |

numDF, numerator degrees of freedom; denDF, denominator degrees of freedom.

Individual was included as a random factor in both models. For the response to soil moisture, the standard deviation of random components were as follows: Individuals = 4.9; residual = 17.1. For the response to VPD, the standard deviation of random components were as follows: Individuals = 5.5; residual = 21.3

Statistically significant values are indicated in bold text.

**Table S5:** ANOVA and AIC results table showing summary statistics for the comparison of the full linear model of recovery of total daily J_s_ (% of maximum J_s_) or G_sf_ (% of maximum G_sf_) as a function of species and season with an interaction with the final model presented in the manuscript.

| **Response Variable** | **Factor** | **AIC** | **D.F.** | **F** | **P** |
| --- | --- | --- | --- | --- | --- |
| **Total daily J_s_** | species*season + (1\|individual) | **162.2** | **7** |  |  |
|  | species + (1\|individual) | **164.9** | **4** | **2.59** | **0.09** |
| G_sf_ | species*season + (1\|individual) | **178.4** | **7** |  |  |
|  | season + (1\|individual) | **177.2** | **3** | **1.45** | **0.27** |

**Table S6:** ANOVA results table showing main effects results of the linear models of Minimum midday and minimum predawn leaf or culm water potential (response variable) as a function of species and season including an interaction.

| **Response Variable** | **Factor** | **D.F.** | **Sum Sq.** | **F** | **P** |
| --- | --- | --- | --- | --- | --- |
| Minimum midday water potential | **Species** | **2** | **17.1** | **59.4** | **0.000** |
|  | **Season** | **1** | **5.8** | **40.3** | **0.000** |
|  | **Species:season** | **2** | **4.5** | **15.6** | **0.000** |
|  | Residuals | 24 | 3.4 |  |  |
| Minimum predawn water potential | **Species** | **2** | **30.5** | **47.5** | **0.000** |
|  | **Season** | **1** | **9.0** | **28.0** | **0.000** |
|  | **Species:season** | **2** | **4.6** | **7.2** | **0.004** |
|  | Residuals | 24 | 7.7 |  |  |

**Supplementary Figures with Captions**


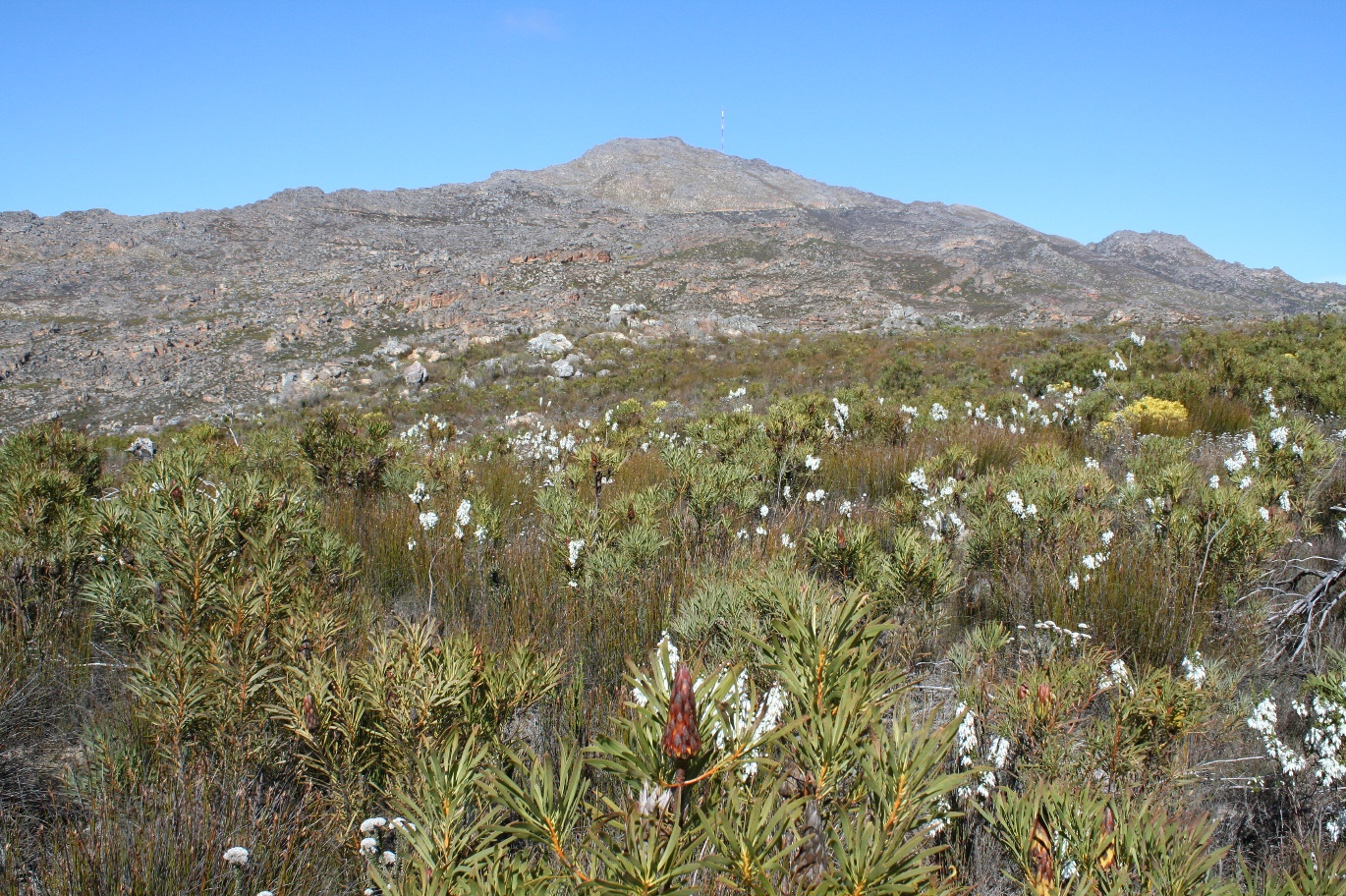

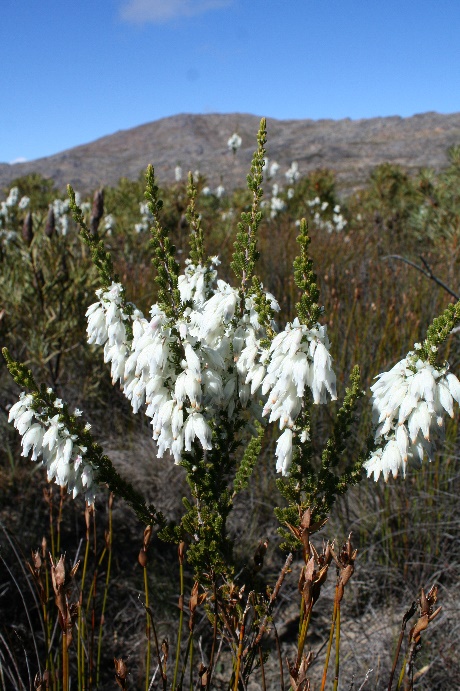

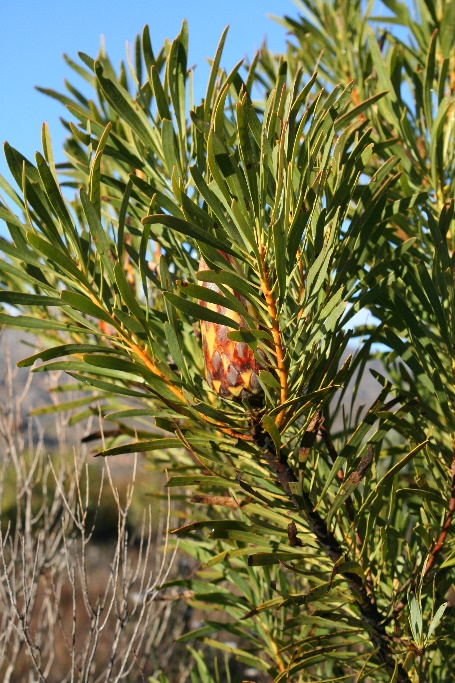

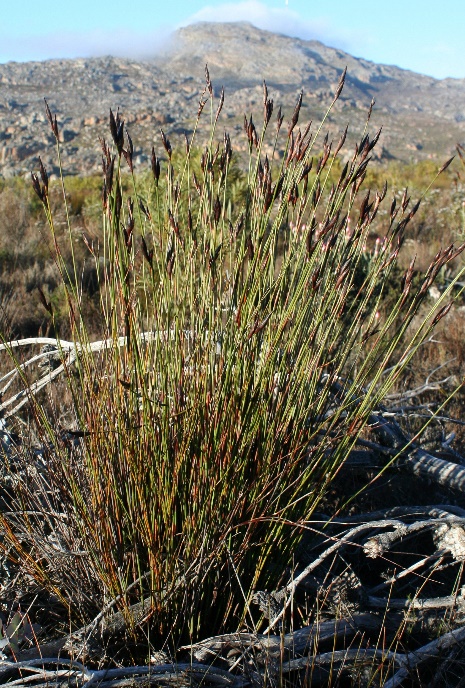


**Figure S1:** The three study species (*Erica monsoniana*, *Protea repens* and *Cannomois congesta*), representative of the three major growth forms of mountain fynbos (i.e., ericoid, proteoid and restioid). The bottom panel shows the Jonaskop study site.


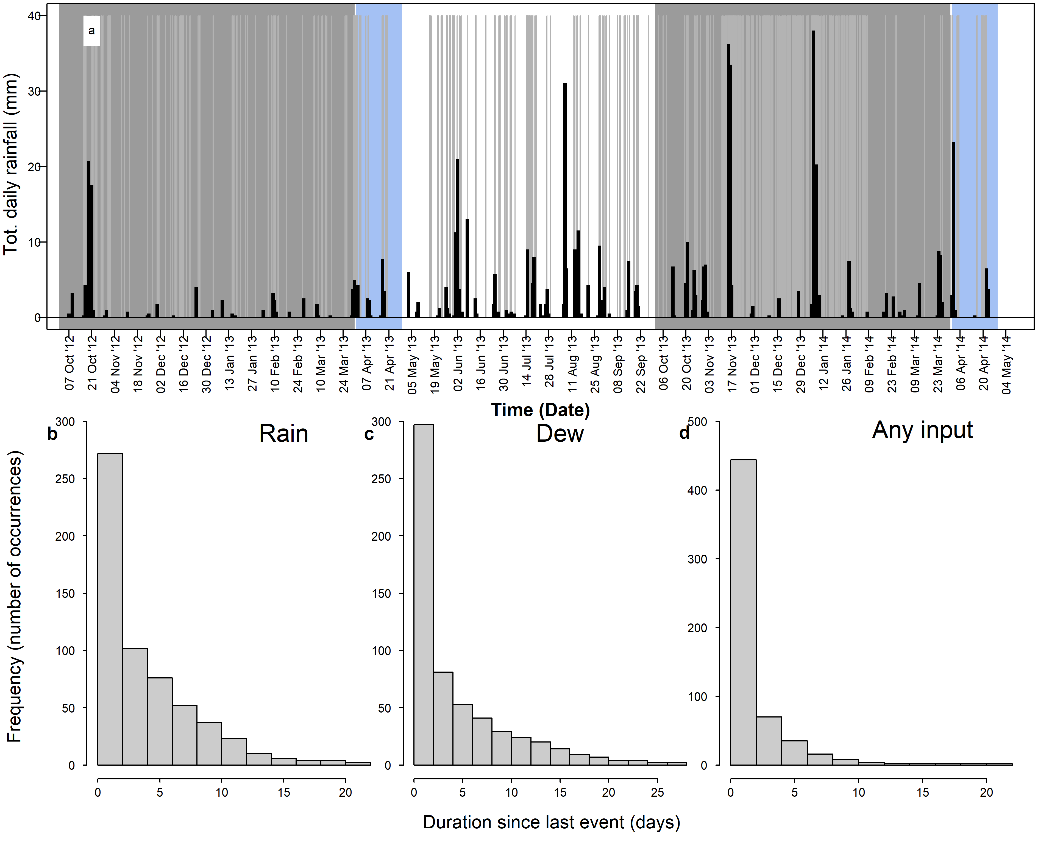


**Figure S2:** Frequency of rain (black bars in panel a), dew (light grey lines in panel a) and all moisture inputs recorded at the study site for the duration of the study period.


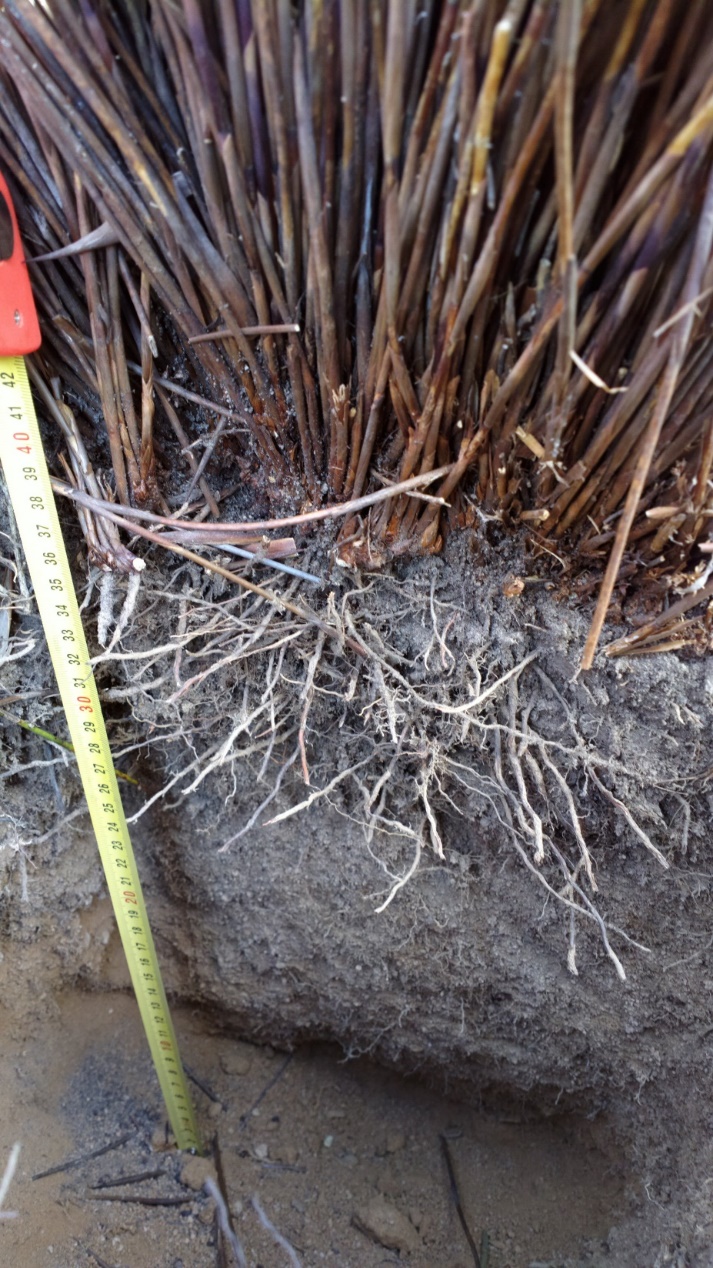


**Figure S3:** Excavations of *Cannomois congesta* individuals showed that individuals are rooted in shallow soil layers (< 40 cm).
